# P-638. Impact of Methicillin-Resistant *Staphylococcus aureus* (MRSA) Nasal Screening on Antimicrobial De-escalation in Patients Admitted with Community-Acquired Pneumonia

**DOI:** 10.1093/ofid/ofae631.835

**Published:** 2025-01-29

**Authors:** Abhishek Deshpande, Rebecca Schulte, Ramara E Walker, Andrea Pallotta, Ming Wang, Michael Klompas, Sarah Haessler, Michael Rothberg

**Affiliations:** Cleveland Clinic, Cleveland, Ohio; Cleveland Clinic, Cleveland, Ohio; Cleveland Clinic, Cleveland, Ohio; Cleveland Clinic, Cleveland, Ohio; Case Western Reserve University, Cleveland, Ohio; Harvard Medical School and Harvard Pilgrim Health Care Institute, Boston, Massachusetts; University of Massachusetts Medical School-Baystate, Springfield, MA; Cleveland Clinic, Cleveland, Ohio

## Abstract

**Background:**

Methicillin-resistant *Staphylococcus aureus* (MRSA) is an important yet uncommon cause of inpatient community-acquired pneumonia (CAP). While the American Thoracic Society/Infectious Diseases Society of America (ATS/IDSA) guidelines recommend rapid MRSA nasal screening to guide anti-MRSA antibiotic (abx) therapy when MRSA CAP is suspected, guideline adherence and its impact on abx use is unclear. We aimed to assess the prevalence of MRSA screening and its effect on abx de-escalation in inpatients with CAP.

**Methods:**

This retrospective cohort study analyzed adult admissions for CAP across 12 U.S. hospitals from November 2022 - January 2024. Anti-MRSA abx utilization and MRSA screening was assessed in both ICU and non-ICU patients. De-escalation was defined as stopping all anti-MRSA agents within 48 hours of the first dose. We compared abx de-escalation rates of patients started on empiric anti-MRSA between those screened vs those not screened. Propensity score adjustment methods were employed to assess the association between MRSA screening and anti-MRSA antibiotic de-escalation, calculating the odds ratio (OR) for de-escalation. Positive and negative predictive values of MRSA screening were also calculated.

**Results:**

Of 12,232 CAP hospitalizations, 1886 were admitted to ICU and the rest to medical wards. Thirty percent of non-ICU and 68% of ICU patients were started on anti-MRSA antibiotics within 48 hours of admission. CAP patients started on empiric anti-MRSA antibiotics were significantly more likely than those not started on them to be screened (95% vs. 74% in ICU and 76% vs. 19% outside ICU, p< 0.001 for both). Patients with a negative MRSA screen were more often de-escalated within 48 hours than those with no screening outside the ICU (84% vs. 71%, p< 0.001) and in the ICU (82% vs. 55%, p< 0.001). In propensity-adjusted analysis, screened patients had significantly greater odds of de-escalation compared to those not screened (non-ICU OR: 2.10 95% CI 1.85-2.38; ICU OR: 3.65 95% CI 3.00-4.45). The positive and negative predictive values of MRSA screening were 3.33% and 99.86% in non-ICU, and 6.83% and 99.78% in ICU, respectively.

**Conclusion:**

MRSA screening was strongly associated with abx de-escalation, especially in the ICU. A negative screen had a very high negative predictive value.

**Disclosures:**

**Abhishek Deshpande, MD, PhD**, AHRQ: Grant/Research Support|Clorox Healthcare: Grant/Research Support **Andrea Pallotta, PharmD**, Astra Zeneca: Advisor/Consultant|Viiv: Advisor/Consultant **Michael Klompas, MD, MPH**, AHRQ: Grant/Research Support|CDC: Grant/Research Support|UpToDate: Royalties **Michael Rothberg, MD, MPH**, Blue Cross Blue Shield: Advisor/Consultant|CRISPER: Stocks/Bonds (Public Company)|Moderna: Stocks/Bonds (Public Company)

